# Effect of Short-Term Thyroxine Administration on Energy Metabolism and Mitochondrial Efficiency in Humans

**DOI:** 10.1371/journal.pone.0040837

**Published:** 2012-07-26

**Authors:** Darcy L. Johannsen, Jose E. Galgani, Neil M. Johannsen, Zhengyu Zhang, Jeffrey D. Covington, Eric Ravussin

**Affiliations:** 1 Pennington Biomedical Research Center, Baton Rouge, Louisiana, United States of America; 2 Department of Nutrition, Faculty of Medicine, University of Chile, Santiago, Chile; Mayo Clinic, United States of America

## Abstract

The physiologic effects of triiodothyronine (T3) on metabolic rate are well-documented; however, the effects of thyroxine (T4) are less clear despite its wide-spread use to treat thyroid-related disorders and other non-thyroidal conditions. Here, we investigated the effects of acute (3-day) T4 supplementation on energy expenditure at rest and during incremental exercise. Furthermore, we used a combination of *in situ* and *in vitro* approaches to measure skeletal muscle metabolism before and after T4 treatment. Ten healthy, euthyroid males were given 200 µg T4 (levothyroxine) per day for 3 days. Energy expenditure was measured at rest and during exercise by indirect calorimetry, and skeletal muscle mitochondrial function was assessed by *in situ* ATP flux (^31^P MRS) and *in vitro* respiratory control ratio (RCR, state 3/state 4 rate of oxygen uptake using a Clark-type electrode) before and after acute T4 treatment. Thyroxine had a subtle effect on resting metabolic rate, increasing it by 4% (p = 0.059) without a change in resting ATP demand (i.e., ATP flux) of the *vastus lateralis*. Exercise efficiency did not change with T4 treatment. The maximal capacity to produce ATP (state 3 respiration) and the coupled state of the mitochondria (RCR) were reduced by approximately 30% with T4 (p = 0.057 and p = 0.04, respectively). Together, the results suggest that T4, although less metabolically active than T3, reduces skeletal muscle efficiency and modestly increases resting metabolism even after short-term supplementation. Our findings may be clinically relevant given the expanding application of T4 to treat non-thyroidal conditions such as obesity and weight loss.

## Introduction

Thyroid hormone circulates in human plasma in two primary forms, triiodothyronine (T3) and thyroxine (T4), the lesser metabolically active of the two. The daily production of T4 is about 100 µg, all of which is produced by the thyroid gland. The daily production of T3 is about 30 µg, of which about 20 percent is produced by the thyroid gland and 80 percent by deiodination of thyroxine in extrathyroidal tissues [1]. The physiologic effects of elevated T3 are well-documented and include reduced insulin sensitivity [2], loss of both fat and lean tissue [3], increased resting [4] and exercise energy expenditure [3], elevated heart rate and feelings of nervousness and palpitations [2].

The stimulatory effects of T3 are exerted through the action of the hormone on nuclear T3 receptors and through nongenomic mechanisms including ion pump activity (Ca^2+^-ATPase and Na^+^/K^+^-ATPase) [5], futile cycling [6], [7] and mitochondrial biogenesis [8]. Because of the significant side effects, T3 is very rarely used as a clinical treatment and T4 has been identified as the hormone of choice to treat hypothyroid disorders [9]. Although attempts have been made to combine T3 and T4 to treat hypothyroidism [10], [11], complaints of palpitations, irritability, dizziness, tremor, perspiration and shortness of breath have slowed this endeavor. Furthermore, there is increasing interest on the use of T4 in non-thyroidal conditions such as coronary artery disease, cardiomyopathy, acute renal failure, severe burn injury, and caloric deprivation [12]. Thus, the use of T4 to treat widespread conditions outside of hypothyroidism may be on the horizon. The metabolic effects of T4 supplementation are not trivial, however. In hypothyroid patients who were chronically treated with T4, modifying the dose even slightly was associated with significant changes in resting energy expenditure [13]. The maintenance of constant T3 levels suggested that energy expenditure was being altered by T4-dependent pathways [13]. Despite its potentially large-scale clinical use, there are relatively few studies on the metabolic effects and mechanism of action of T4 [14], [15], [16], particularly in euthyroid individuals [17], [18].

Conversely, the mechanism of action of T3 has been fairly well established. A single daily dose of 75 µg T3 in five healthy men for 14 days was found to up-regulate genes involved in glucose and lipid metabolism, protein synthesis, transcriptional control, signal transduction, and mitochondrial energy metabolism including increased uncoupling protein 3 [19]. Furthermore, the *in situ* mechanism of T3 was in part described by Lebon et al [4], who investigated the effect of short-term (3 days) T3 supplementation on skeletal muscle mitochondrial metabolism using *in vivo* spectroscopic techniques. They found that TCA cycle activity was increased by 70% but ATP synthesis was unchanged, suggesting reduced mitochondrial coupling (i.e., efficiency) with T3 treatment. It is thought that T4 works in a similar manner, but this has yet to be determined. Additionally, no previous study has investigated the acute metabolic effects of T4 supplementation, despite its known effects on metabolism over a longer duration [17]. Therefore, the purpose of the study was to investigate the acute effects of T4 supplementation on energy expenditure and mitochondrial activity in healthy, euthyroid subjects. We hypothesized that similar to T3, increasing T4 above normal baseline levels would increase energy expenditure at rest and during exercise and would affect mitochondrial activity by reducing coupling efficiency.

## Results

Subjects were age 25.0±4.6 years old and had an average BMI of 23.3±2.0 kg/m^2^ (19.6–26.7). Average fat-free mass (FFM) was 60.9±3.3 kg and FM was 13.5±4.2 kg (18.0±4.8% fat, 9.6–26.7%). All subjects had thyroid hormone levels within normal limits at screening [T3, 113 (94–137) ng/dl; TSH, 2.08 (1.17–3.08) µU/ml]. Total and free T4 concentrations increased with T4 treatment, T3 did not change, and TSH decreased, suggesting suppressed thyroid stimulation. Thyroid binding capacity was unchanged (Table 1). Body weight did not change during the study (Table 2).

**Table 1:** Thyroid profile before (Day 1) and after (Day 4) 3 days of T4 supplementation (mean ± SD).

	Day 1	Day 4	*P* value
**T3 (ng/dl)**	111±15	109±12	0.68
**TSH (µU/ml)**	1.82±0.95	0.87±0.35	**0.005**
**T4 (µg/dl)**	7.1±1.0	10.0±0.7	**0.003**
**Free T4 (ng/dl)**	1.18±0.13	1.56±0.13	**<0.001**
**Thyroid binding capacity (µg/dl)**	17.0±3.8	17.2±3.7	0.57

**Table 2:** Metabolic rate (RMR), respiratory quotient (RQ) and vital signs during rest before (Day 1) and after (Day 4) 3 days of T4 treatment (mean ± SD).

	Day 1	Day 4	*P* value
**Body Weight (kg)**	75.0±4.7	75.0±4.8	1.00
**RMR (kcal/d)**	1612±142	1672±129†	**0.059**
**Resting RQ**	0.83±0.03	0.85±0.03	**0.02**
**Heart Rate (bpm)**	57±9	59±8	0.38
**Blood Pressure** **(mm Hg)**			
* Systolic*	122±9	121±6	0.91
* Diastolic*	83±6	83±6	0.84

Resting metabolic rate increased by 60±109 kcal/day from day 1 to day 4, a 4.0±6.8% increase (p = 0.06) and was quite variable among subjects (−119 to +252 kcal/day). Resting respiratory quotient was significantly higher after T4 supplementation, and blood pressure and heart rate were unchanged (Table 2). Table 3 shows the effect of T4 supplementation on heart rate and parameters of exercise efficiency during an incremental exercise test. As expected, heart rate, oxygen uptake (VO_2_), and energy expenditure increased with increasing workloads (p<0.001); however, none of the exercise parameters differed by visit (day 1 vs. day 4) and the responses for each visit across all workloads were similar (interaction p>0.30 for all variables). Delta efficiency was not significantly different at baseline and after 3 days of T4 treatment (27.1±1.5% and 26.1±1.5%, respectively; p = 0.14).

**Table 3:** Exercise metabolic data before and after 3 days of T4 treatment (mean ± SD).

	Workload					*P value*	
	0 Watts	35 Watts	70 Watts	105 Watts	140 Watts	Visit	Interaction
**Heart Rate (bts/min)**							
Day 1	87.0±18.2	95.1±18.6	113.4±22.8	136.1±26.7	158.8±27.0		
Day 4	83.8±13.5	93.6±14.3	110.3±18.4	134.4±24.7	158.0±26.3	**0.27**	**0.33**
**VO_2_ (L/min)**							
Day 1	0.58±0.11	0.81±0.07	1.13±0.09	1.48±0.07	1.91±0.08		
Day 4	0.60±0.09	0.80±0.07	1.12±0.07	1.50±0.10	1.93±0.09	**0.66**	**0.42**
**RER**							
Day 1	0.84±0.03	0.85±0.05	0.93±0.06	1.00±0.08	1.06±0.12		
Day 4	0.85±0.07	0.88±0.07	0.93±0.08	1.01±0.10	1.08±0.10	**0.26**	**0.72**
**Energy Expenditure (kcal/min)**							
Day 1	2.79±0.54	3.93±0.33	5.54±0.42	7.37±0.35	9.50±0.40		
Day 4	2.89±0.42	3.88±0.32	5.52±0.34	7.46±0.45	9.65±0.43	**0.61**	**0.49**
**Gross Efficiency (%)**							
Day 1		12.8±1.1	18.2±1.4	20.5±1.0	21.1±0.9		
Day 4		13.0±1.0	18.2±1.1	20.2±1.2	20.8±0.9	**0.79**	**0.51**
**Work Efficiency (%)**							
Day 1		25.4±1.7	28.0±1.6	27.9±2.0	26.6±1.4		
Day 4		24.4±1.3	27.2±1.9	26.8±2.2	25.7±1.3	**0.14**	**0.99**
**Delta Efficiency (%)**							
Day 1			31.5±3.5	28.0±4.4	23.6±2.1		
Day 4			31.0±4.7	26.9±5.6	23.2±2.5	**0.30**	**0.98**

*P values* for the main effect of visit and the visit x exercise workload interaction are from two-way repeated measures ANOVA. VO_2_, volume of oxygen consumed in liters of O_2_ per minute; RER, respiratory exchange ratio.

**Figure 1:**
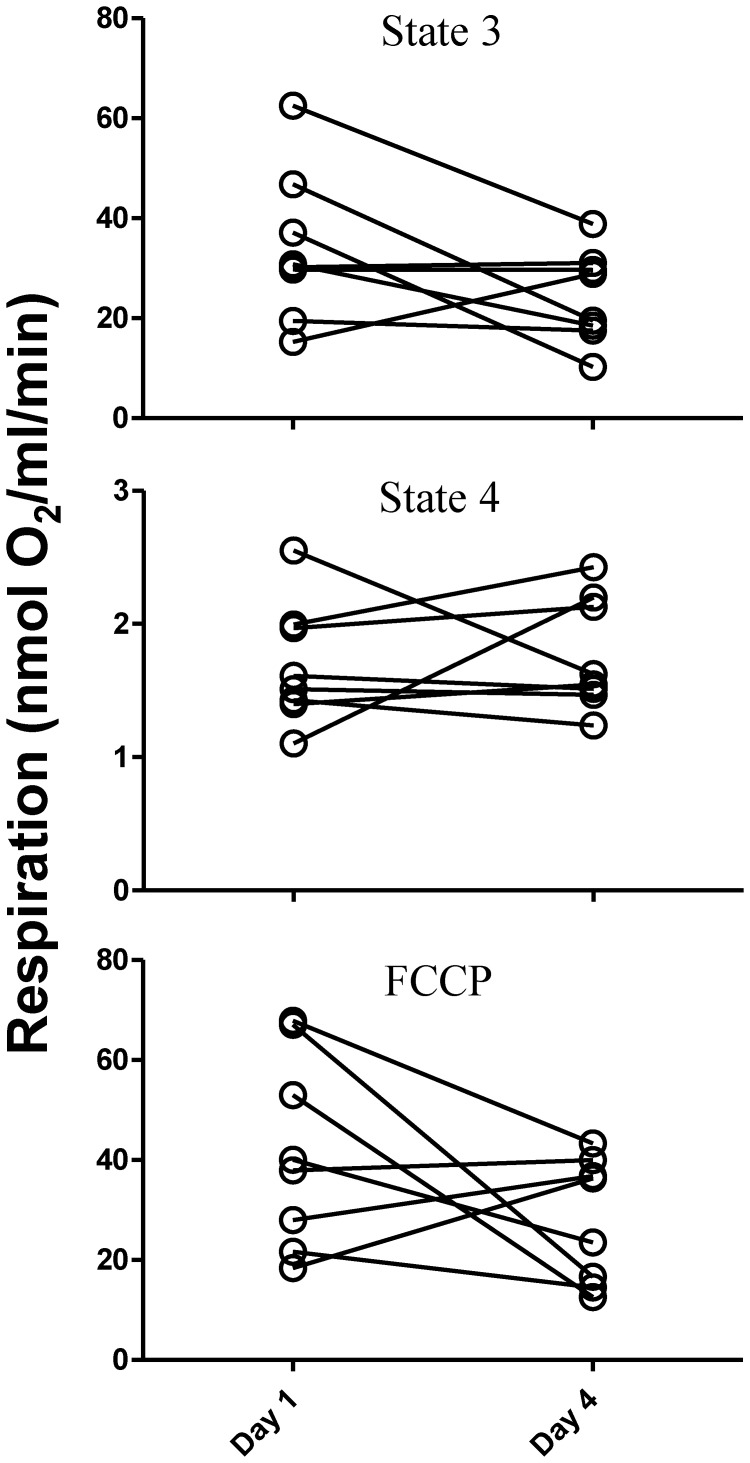
State 3 and state 4 respiration and FCCP-induced maximal respiration of mitochondria isolated from the *vastus lateralis* before and after 3 days of T4 treatment (n = 8). State 3, or ADP-stimulated respiration was decreased (p = 0.057) with no change in state 4 (leak-dependent) respiration (p = 0.37). Maximal respiration induced by the addition of the uncoupler FCCP also tended to decrease with T4 treatment (p = 0.07).

At the tissue level, *in situ* ATP flux of the *vastus lateralis* (^31^P MRS) did not change with T4 treatment (5.3±0.6 to 5.2±0.6 µM ATP/sec, p = 0.52, day 1 to day 4, respectively). In isolated mitochondria, maximal ADP-coupled (state 3) respiration tended to be reduced following T4 treatment; from 34±15 to 24±9 nmol O_2_/ml/min, respectively (p = 0.057). However, leak-mediated respiration (state 4) was unchanged (1.7±0.5 to 1.8±0.4 nmol O_2_/ml/min, p = 0.37) (Figure 1). Therefore, the respiratory control ratio (RCR; ratio of state 3/state 4 =  an index of mitochondrial coupling) was reduced after T4 treatment from 21±9 to 15±6 (p = 0.04) but was due to decreased ADP-coupled respiration rather than increased proton leak. Maximal respiration induced by FCCP decreased by 33% after T4 treatment, from 42±19 nmol O_2_/ml/min on day 1 to 28±13 nmol O_2_/ml/min on day 4 (p = 0.07) (Figure 1). The reductions in ADP-coupled respiration and FCCP-stimulated maximal respiration following T4 treatment were highly correlated (r = 0.96, p<0.001), suggesting that the declines in phosphorylating and maximal mitochondrial respiration were a true effect of T4 and not measurement artifact.

To investigate whether uncoupling protein may underlie this change, we measured UCP3 expression before and after T4 supplementation. UCP3 expression increased 31% from day 1 to day 4 however the change was not statistically significant (1.05±0.48 to 1.38±0.75 AU, p = 0.28). Mitochondrial content (mtDNA, the average of the ratio of ND1 and ND4 to 18S genes) did not change from day 1 to day 4 (1.29±0.34 vs. 1.25±0.67 respectively, p = 0.85).

**Figure 2:**
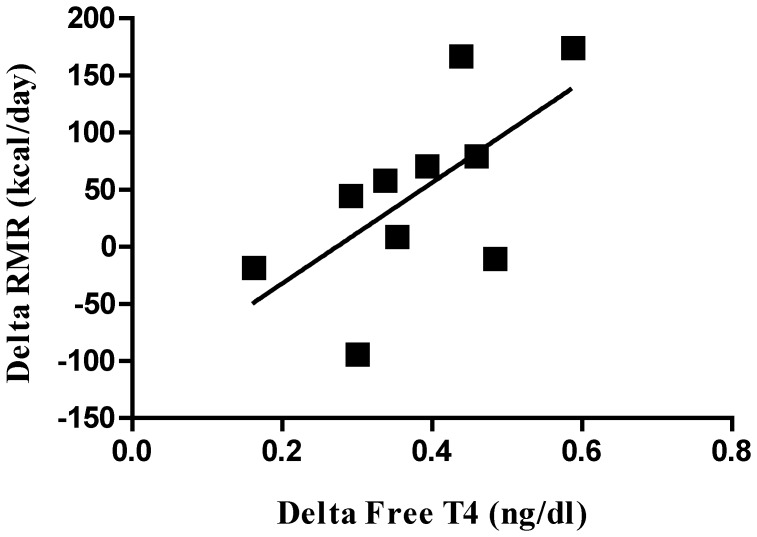
Relationship between the change in resting metabolic rate and the change in free T4 after 3 days of T4 treatment, after controlling for baseline free T4 levels. r = 0.61, p = 0.06.

Due to the considerable variability in response to T4 supplementation among subjects, we investigated whether changes in free T4 levels were associated with changes in energy expenditure or mitochondrial coupling. To factor out the influence of baseline levels, we included baseline free T4 in the models. A larger increase in free T4 after supplementation (day 4-day 1) tended to be associated with a larger increase in RMR (r = 0.61, p = 0.06) (Figure 2) but not with delta exercise efficiency (r = −0.19, p = 0.59), change in ATP flux (r = −0.05, p = 0.90), or change in mitochondrial coupling (r = −0.17, p = 0.71). We also examined whether percent body fat influenced the response to T4. Subjects with higher percent body fat had lower free T4 levels at baseline (r = −0.62, p = 0.056), had an increase in FCCP-induced maximal mitochondrial respiration after T4 supplementation (r = 0.74, p = 0.03) and a trend for increased ADP-linked respiration (r = 0.62, p = 0.10).

## Discussion

The physiologic effects of elevated T3 levels have been well characterized [2], [3] and the effects on energy expenditure are thought to be partly mediated through uncoupling of skeletal muscle mitochondria [4]. Because of its potent side effects including nervousness, irritability, tremors and palpitations, the use of T3 as a hormone replacement therapy is limited, making the less metabolically active T4 the treatment of choice [9]. Despite its widespread use and recent application outside the realm of thyroid disorders [12], the effects of T4 on energy metabolism have not been well described and are contradictory, with one study showing no change [20] and others showing increased energy expenditure [17], [21]. Furthermore, the mechanism by which T4 may increase metabolic rate has not been investigated.

We sought to determine the effect of acute (3 days) T4 treatment, mimicking an earlier study on T3 supplementation, on whole-body resting and exercise energy expenditure and skeletal muscle metabolism *in situ* and *in vitro*. We found a subtle increase in RMR of 4%, not quite reaching statistical significance. An earlier study in euthyroid, healthy men [17] reported a similar (4%) albeit significant increase in sleeping energy expenditure and an increase in heart rate during 3 weeks of thyroxine supplementation (150–200 µg daily). Since 80% of T3 is formed by the extra-thyroidal deiodination of T4, increasing T4 levels may be expected to also increase T3 levels. The lack of any change in circulating T3 in our study and others [17] suggests that the effect on RMR was independent of T3, and indicates that the level of T3 is likely regulated by deiodinase activity in individuals without thyroid disorders. It is possible that in euthyroid individuals, a larger part of excess T4 is converted to inactive reverse T3 (rT3), thereby conserving normal T3 levels; however, we did not have rT3 data available to substantiate that speculation. The maintenance of normal T3 may also be due to the large drop in TSH, suppressing endogenous T3 production by the thyroid gland.

Whereas previous reports [17] found no change in RQ with T4 treatment, we observed a significant increase in resting RQ indicating a preference for carbohydrate as a fuel source instead of fat. The increase in RQ in our study may have been influenced by the macronutrient composition of the diet provided to them (FQ of 0.92) instead of a true effect of T4 treatment *per se*, particularly in individuals whose usual diet was lower in CHO. Alternatively, the increase in RQ could have been a consequence of a positive energy balance in some individuals. Since we did not measure their dietary intake prior to metabolic assessment and energy balance during the intervention, we cannot ascertain how important was the diet, energy balance or T4 treatment in the increase in RQ.

Little attempt has been made to investigate the changes in exercise efficiency with thyroid hormone administration. One recent study reported no difference in body weight and body composition, resting energy expenditure, respiratory exchange ratio, or efficiency of muscular work during 3, 6 and 12 months of thyroxine therapy in euthyroid women with benign thyroid nodules [20]. The average dose was 1.5 µg·kg^−1^·day^−1^L-T4 (around 93 µg/day), which caused a significant decrease in circulating TSH and an increase in free T4 (FT4), in line with the thyroid-suppressing effects of thyroxine. Exercise economy at fixed workloads of 0.5, 0.66, and 1 Watt/kg body weight applied for 3, 10, and 20 minutes, respectively, were similar at baseline and after 12 months of T4 treatment. Similarly, we found no differences in exercise efficiency at any given workload with T4. Although mitochondrial uncoupling was greater after 3 days of T4 supplementation, changes in exercise efficiency were not observed suggesting disconnect between maximally- stimulated mitochondrial respiration and submaximal, whole-body exercise efficiency.

Despite the subtle effect on RMR and lack of effect on exercise efficiency, 3 days of T4 treatment was associated with a decrease in mitochondrial efficiency, specifically a reduction in state 3 respiration by nearly 30%. State 3 is the maximal ADP-coupled respiratory capacity of the mitochondria, and a decrease suggests a reduced functional capacity of mitochondria to produce ATP. The oxygen consumption of isolated mitochondria using pyruvate and malate as substrates represents Complex I-driven respiration involving the TCA cycle to generate NAD, activity of other complexes of the ETC including the ATPsynthase, mitochondrial membrane potential, and the adenine nucleotide transporter (ANT) [22]. Thus, it is difficult to ascertain which component is responsible for the decreased state 3 respiration.

The decrease in mitochondrial efficiency occurred in the presence of no change in muscle ATP turnover (i.e., demand). Reduced functional capacity to generate ATP given similar ATP requirements would require additional substrate to flux through the mitochondria in order to meet energy demands. Reduced mitochondrial efficiency has previously been reported with T3 treatment [4], including a 70% increase in skeletal muscle TCA cycle flux and no change in ATP synthesis, which implies that efficiency is reduced due to increased proton leak. However, our data suggest that T4 treatment reduces mitochondrial coupling efficiency through decreased oxidative phosphorylation activity with similar proton leak. We are not able to determine whether the discrepancy is due to different (*in situ* vs. *in vitro*) techniques used, or is a distinct effect of T3 vs. T4 on mitochondrial efficiency. The 30% decrease in efficiency but only 4% reduction in RMR seems contradictory; however, discrepancy between changes in muscle metabolism vs. whole-body metabolism was previously reported with T3 treatment [4], and is probably due to the significant contribution of extra-muscular tissues to whole-body resting metabolism.

Although all subjects were non-obese, percent body fat varied considerably among subjects with a range of 9.6 to 26.7%, and subjects who had a greater percent fat had lower T4 levels at baseline, although none were considered hypothyroid (T4 range at baseline, 6.0–8.9 µ/dl). Interestingly, those with higher percent fat had increased phosphorylating and maximal mitochondrial respiration after T4 supplementation, suggesting an effect of body fat on the response to treatment. Thyroid function has been reported to be altered in obesity previously [23], and in a large population study conducted in euthyroid patients [24], BMI was positively associated with serum TSH and inversely with FT4. Given our small sample size, it is noteworthy that we found a similar association between adiposity and free T4 levels and highlights the potential clinical significance of thyroid function in obesity.

Interpretability of our study is limited by the relatively small number of participants and the short duration of T4 treatment. Furthermore, in this preliminary investigation we included only younger males who were of normal weight. Our results are meant to represent early findings on the *in vitro* and *in vivo* mitochondrial effects of acute thyroxine supplementation in healthy adults. To summarize, our results suggest that T4, although less metabolically active than T3, reduces skeletal muscle efficiency and modestly increases resting metabolism even after short-term supplementation in euthyroid normal-weight males. Our findings may be clinically relevant given the expanding application of T4 to treat non-thyroidal conditions such as obesity and weight loss. Next steps should investigate the effects of longer-duration T4 supplementation on metabolism and mitochondrial activity in a larger sample including overweight or obese males and females.

## Materials and Methods

### Subjects

Ten healthy normal- to mildly over-weight (BMI 20–27 kg/m^2^) male Caucasian subjects were enrolled in this study. We chose to study only young non-obese males to reduce biological variability due to sex, age and obese status. All subjects were non-smoking and not taking any medications affecting body weight or metabolism. Prior to participation, subjects underwent a screening visit where they had their height, weight, and vital signs (blood pressure, heart rate, body temperature) measured, answered questions about their health history, and underwent a standard physical exam and blood draw for measurement of chemistry panel, lipid profile and thyroid function (T3 and TSH).

### Ethics Statement

All subjects gave written informed consent, and the study was approved by the Institutional Review Board of the Pennington Biomedical Research Center.

### Study Design

**Figure 3:**
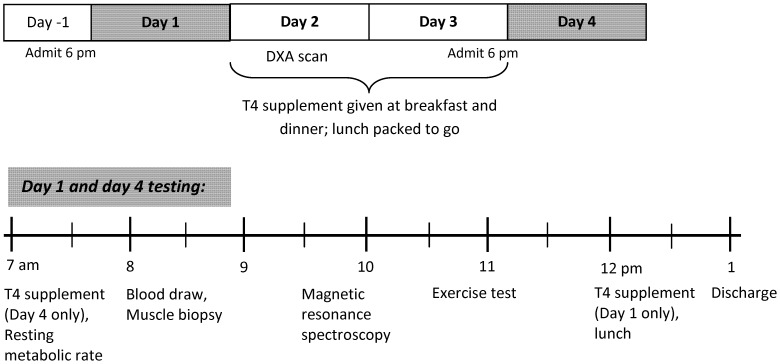
Experimental protocol and timeline of testing procedures.

Participants received a total of 600 µg of T4 over the course of the study, given in 100 µg doses administered approximately every 12 hours. The daily T4 dose was chosen to be in the upper range of usual treatment doses, which varies from 75 to 250 µg/day [9]. The average dose per kg body weight was 2.7±0.2 µg/day. The experimental protocol and timing of testing procedures is outlined in Figure 3. Subjects were admitted to the Inpatient Unit of the Pennington Biomedical Research Center the evening before baseline (day 1) and post-treatment (day 4) testing. After completing baseline testing on day 1, subjects were given their first T4 dose (levothyroxine sodium 100 µg tablet, Sandoz, Princeton, NJ) followed by lunch and were then free to leave the Center. On Days 2 and 3 participants were given 100 µg T4 prior to consuming breakfast and dinner at the Center; lunch was packed to go. Subjects were re-admitted to the Inpatient Unit the evening of day 3. On the morning of day 4, subjects consumed their last thyroxine dose prior to repeating baseline testing. Caloric requirements to maintain energy balance throughout the 4-day study were determined using previously published equations [25]. All meals were prepared by the Metabolic Kitchen and consisted of 55% CHO, 35% fat, and 15% protein.

### Body Composition

Body composition was measured one time during the study, on day 2, by dual energy x-ray absorptiometry (DXA; Hologics, QDA 4500A; Bedford, MA). Fat-free mass (FFM) and fat mass (FM) were calculated from weight and whole-body percent fat.

### Day 1 and Day 4 Testing Procedures

#### Resting metabolic rate

Following a 10-h overnight fast, resting metabolic rate (RMR) was determined by measuring O_2_ consumption and CO_2_ production using a metabolic cart (Deltratrac II, Sensormedics; Yorba Linda, CA). The cart was calibrated before each test with room air and standard calibration gases (4% CO_2_ and 96% O_2_). The first 10 minutes of data were discarded and the last 20 minutes were used to calculate RMR. Resting respiratory quotient (RQ), an index of the mix of energy substrate oxidation, was calculated as the ratio of CO_2_ produced/O_2_ consumed. Blood was drawn after the RMR test on days 1 and 4 for measurement of thyroid hormones.

#### Skeletal muscle ATP turnover

Following the RMR measure, ATP turnover (ATP_flux_) of the *vastus lateralis* was measured by ^31^P magnetic resonance spectroscopy as previously described [26], [27]. Briefly, spectra were acquired during a 5-min baseline rest period and for 16 min while a blood pressure cuff was inflated above the site of interest to 60 mm Hg above systolic pressure. The breakdown of phosphocreatine under anoxic conditions represents mitochondrial ATP demand, i.e., cellular ATP use minus glycolytic ATP supply [28], [29]. Spectra were analyzed using the Advanced Method for Accurate, Robust and Efficient Spectral fitting (AMARES) algorithm in the jMRUI software [30]. Using this method, repeated measures of muscle ATP_flux_ on the same subject (n = 4) agree to within ±5.0%.

#### Mitochondrial coupling

Muscle biopsy was performed at the site of the MRS spectra acquisition using the Bergstrom technique [31]. Immediately following tissue collection, approximately 100–150 mg of whole muscle was minced in 1 ml ice-cold mitochondrial extraction buffer (5 mM MgCl_2_, 100 mM KCl, 40 mM Tris-HCl, 10 mM Tris-Base, 1 mM EDTA, 1 mM ATP, pH 7.5, ice cold) and subjected to a protease digestion (Type XXIV, Sigma Chemical Co., St. Louis, MO) for 7 minutes [32]. Protease digestion was terminated immediately by adding 1 ml of extraction buffer. The tissue was homogenized and centrifuged at 700 g and the supernatant was collected and centrifuged again at 14,000 g to collect a mitochondria pellet. The final pellet was resuspended in Mannitol-Sucrose Solution (220 mM Mannitol, 70 mM Sucrose, 10 mM Tris-HCl, 1 mM EGTA, pH 7.4, ice cold). Mitochondrial protein concentration was determined spectrophotometrically using the bicinchoninic acid (BCA) assay (Thermo Scientific, 23225, Rockford, IL).

Oxygen consumption was measured in isolated mitochondria at 37°C using a Clark-type oxygen electrode (Hansatech Instruments, Norfolk, England). The electrode was surrounded by a temperature-controlled water-jacketed glass chamber to maintain the temperature at 37°C. Mitochondria were suspended at a concentration of 80 µg/400 µl in Wander’s Respiration Buffer and the measurement was carried out in a medium containing 100 mM KCl, 50 mM MOPSO, 10 mM K_2_HPO_4_, 10 mM MgCl_2_, 1 mM EDTA, 20 mM glucose, 5 mM glutamate, 0.2%BSA and pH 7.0. Mitochondrial respiration was initiated by adding the substrates sodium pyruvate (1.25 mM) and malate (1.25 mM). After reaching a stable rate, coupled respiration was initiated by adding ADP (0.375 mM). After determination of the maximal ADP-initiated respiration, oligomycin (1.6 µg/ml) was added to block complex V (ATP synthase) and provide a measure of non-ADP coupled respiration (i.e. leak-induced). Rates of oxygen consumption were expressed as nmol O_2_/ml/min. The respiratory control ratio (RCR) was defined as the rate of ADP-stimulated oxygen consumption (State 3) divided by the rate of leak-induced respiration (State 4). FCCP (3 µM) was added to stop state 4 respiration and induce maximal mitochondrial respiration.

#### Mitochondrial content

Mitochondrial (mt) content was determined as the average of the ratio of ND1 and ND4 (mitochondrial-encoded) to 18S (nuclear-encoded) genes. Briefly, DNA was extracted from the *vastus lateralis* biopsy tissue using the Blood and Tissue DNA extraction kit (Qiagen) per manufacturer’s protocol. mtDNA was determined using qPCR methods measured on a 7900 Sequence Detection System (Applied Biosystems) using Assays on Demand (ND1: Hs02596873_s1; ND4: Hs02596876_g1; 18S: Hs99999901_s1). qPCR expression was quantified using a standard curve of known DNA concentrations.

#### Uncoupling protein 3 expression

RNA from *vastus lateralis* tissue that had been snap- frozen and stored in liquid nitrogen was extracted using the miRNEasy mini kit (Qiagen) per manufacturer’s protocol. cDNA was made using the High Capacity cDNA Reverse Transcriptase Kit (Applied Biosystems). Gene expression was measured on a 7900 Sequence Detection System using Assays on Demand (UCP3: Hs01106052_m1; RPLP0: 99999902_m1). Expression of UCP3 was quantified using a standard curve of known cDNA loading concentration and normalized to the expression of the housekeeping gene Ribosomal Protein, Large Protein 0 (RPLP0).

#### Exercise efficiency

A week prior to the exercise efficiency test, participants were familiarized with the protocol and equipment to reduce the test-retest variability. Non-resting metabolic efficiency was determined by measuring energy expenditure (EE) during an incremental exercise test on an electronically-braked cycle ergometer [33] (Lode, Groningen, The Netherlands). Briefly, after a 10-min warm-up period at 0 Watts (W, no resistance), subjects cycled for 6 min at 0 W to measure respiratory gas exchange during ‘unloaded’ exercise. Then, participants underwent an incremental exercise test for 6 min per stage at 35 W, 70 W, 105 W and 140 W while maintaining a constant pedal rate of 60 RPM. Stages were separated by 6 min of rest. CO_2_ and O_2_ were measured during the entire test (TruOne 2400, ParvoMedics, Inc. Sandy, Utah) with the average of the last 2 min of each stage (steady state) used to determine energy expenditure and substrate utilization. Exercise efficiency was expressed in several ways for this study: gross, work, and delta efficiency. Gross efficiency was defined as the workload divided by energy expenditure. Work efficiency was calculated as the workload divided by the estimated load-free (y-intercept) subtracted energy expenditure at a given workload. The relative cost of performing incrementally increasing work (delta efficiency) was calculated as the inverse of the slope (derivative of dE/dW) for energy expenditure (E) and work rate (W) across workloads. Fitness level was not used as an exclusion criterion and we expect that our participants were of varied fitness levels. As such, we anticipated that energy expenditure at the higher workloads in the lesser fit participants may be influenced by anaerobic metabolism. To account for this, the caloric equivalents for oxygen were clamped to those of an RER  = 1.00 suggestive of 100% carbohydrate oxidation.

### Statistical Analysis

Data are expressed as mean ± SD or mean (range) and the level of significance for all statistical tests was set at p<0.05. Analyses were performed using JMP version 7.0.1 (SAS Institute, Cary, NC). Differences in body weight, thyroid hormones, heart rate, blood pressure, and mitochondrial bioenergetics before and after thyroxine treatment were analyzed using paired t-tests. Differences in RMR, resting RQ, and delta efficiency before and after T4 were determined by one- and two-way repeated measures ANOVA with post-hoc analysis using Student’s t test. All data were found to be normally distributed; therefore Pearson correlations were performed on variables of interest. Associations between the change in free T4 level (day 4 - day 1) and the change in RMR, delta efficiency, tissue ATP flux, and mitochondrial coupling were analyzed by multiple linear regression controlling for baseline (day 1) free T4.
